# Direct Proof of the Reversible Dissolution/Deposition of Mn^2+^/Mn^4+^ for Mild‐Acid Zn‐MnO_2_ Batteries with Porous Carbon Interlayers

**DOI:** 10.1002/advs.202003714

**Published:** 2021-02-01

**Authors:** Hyeonseok Moon, Kwang‐Ho Ha, Yuwon Park, Jungho Lee, Mi‐Sook Kwon, Jungwoo Lim, Min‐Ho Lee, Dong‐Hyun Kim, Jin H. Choi, Jeong‐Hee Choi, Kyu Tae Lee

**Affiliations:** ^1^ School of Chemical and Biological Engineering Institute of Chemical Processes Seoul National University 1 Gwanak‐ro Gwanak‐gu Seoul 08826 Republic of Korea; ^2^ Next Generation Battery Research Center Korea Electrotechnology Research Institute Bulmosan‐ro 10beon‐gil, Seongsan‐gu Changwon‐si Gyeongsangnam‐do 51543 Republic of Korea; ^3^ Korea Electric Power Corporation Research Institute 105 Munji‐Ro Yuseong‐Gu Daejeon 34056 Republic of Korea

**Keywords:** aqueous batteries, mild acid electrolytes, porous carbon interlayers, reaction mechanisms, Zn‐MnO_2_ batteries

## Abstract

Mild‐acid Zn‐MnO_2_ batteries have been considered a promising alternative to Li‐ion batteries for large scale energy storage systems because of their high safety. There have been remarkable improvements in the electrochemical performance of Zn‐MnO_2_ batteries, although the reaction mechanism of the MnO_2_ cathode is not fully understood and still remains controversial. Herein, the reversible dissolution/deposition (Mn^2+^/Mn^4+^) mechanism of the MnO_2_ cathode through a 2e^−^ reaction is directly evidenced using solution‐based analyses, including electron spin resonance spectroscopy and the designed electrochemical experiments. Solid MnO_2_ (Mn^4+^) is reduced into Mn^2+^ (aq) dissolved in the electrolyte during discharge. Mn^2+^ ions are then deposited on the cathode surface in the form of the mixture of the poorly crystalline Zn‐containing MnO_2_ compounds through two‐step reactions during charge. Moreover, the failure mechanism of mild‐acid Zn‐MnO_2_ batteries is elucidated in terms of the loss of electrochemically active Mn^2+^. In this regard, a porous carbon interlayer is introduced to entrap the dissolved Mn^2+^ ions. The carbon interlayer suppresses the loss of Mn^2+^ during cycling, resulting in the excellent electrochemical performance of pouch‐type Zn‐MnO_2_ cells, such as negligible capacity fading over 100 cycles. These findings provide fundamental insights into strategies to improve the electrochemical performance of aqueous Zn‐MnO_2_ batteries.

## Graphical abstract

The reversible dissolution/deposition (Mn^2+^/Mn^4+^) mechanism of the MnO_2_ cathode for mild‐acid Zn‐MnO_2_ batteries is directly evidenced using ex situ electron spin resonance spectroscopy and the designed electrochemical experiments. Based on the reaction mechanism, a porous carbon interlayer is introduced to suppress the loss of electrochemically active Mn^2+^ ions during cycling, leading to the excellent electrochemical performance of aqueous Zn‐MnO_2_ batteries.

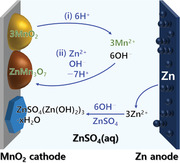

## 1 Introduction

Environmental concerns such as climate change have become one of the global issues that should be overcome for the sustainable development of countries around the world. For this reason, distributed power sources and smart grids, which use large scale energy storage systems (ESSs), have been considered highly promising technologies. Li‐ion batteries have attracted attention as promising power sources for ESSs because of their excellent electrochemical performance, including high energy density and stable cycle performance. Several ESS‐related fire accidents, however, have been reported in the last few years. As a result, recent energy storage systems require not only high energy density but also high safety.^[^ ^1^, ^2^, ^3^, ^4^, ^5^ ^]^ In this regard, many efforts have focused on the development of safe aqueous batteries, such as aqueous Zn‐based batteries,^[^ ^6^, ^7^, ^8^, ^9^ ^]^ aqueous Li and Na‐ion batteries,^[^ ^10^ ^]^ and redox flow batteries,^[^ ^11^ ^]^ because they contain no flammable organic solvents. In particular, mild‐acid Zn‐MnO_2_ batteries have been considered a promising alternative to Li‐ion batteries because of their low cost.^[^ ^12^, ^13^, ^14^ ^]^

Many research groups reported the promising electrochemical performance of mild‐acid Zn‐MnO_2_ batteries; however, the reaction mechanism of MnO_2_ cathode was not fully understood and still remains controversial.^[^ ^15^, ^16^, ^17^, ^18^, ^19^, ^20^, ^21^, ^22^, ^23^, ^24^, ^25^, ^26^, ^27^, ^28^, ^29^, ^30^, ^31^ ^]^ For example, the conversion mechanism suggested the formation of MnOOH during discharge,^[^ ^15^, ^16^, ^17^ ^]^ whereas the intercalation mechanism showed the insertion of Zn^2+^ and/or H^+^ into MnO_2_ during discharge.^[^ ^18^, ^19^, ^20^, ^21^, ^22^, ^23^, ^24^, ^25^, ^26^, ^27^, ^28^ ^]^ The combined conversion/intercalation mechanism was also recently introduced.^[^ ^29^, ^30^, ^31^ ^]^ However, the dissolution and deposition mechanism of Mn^2+^/Mn^4+^ at the MnO_2_ cathode for mild acidic electrolytes has not been demonstrated with direct evidence,^[^ ^32^, ^33^, ^34^ ^]^ despite the fact that no controversy has arisen with respect to the reaction mechanism of Mn^2+^/Mn^4+^ for highly acidic electrolytes.^[^ ^35^ ^]^ Moreover, few reports have considered the possibility of an irreversible reaction mechanism for the initial cycle, in spite of the fact that the initial and subsequent cycles showed different voltage profiles.^[^ ^23^ ^]^ The ambiguity of these reaction mechanisms is attributed to not only the complexity of the reaction mechanism but also the limitation of analytical tools. Solution‐based analyses were rarely considered to demonstrate the reaction mechanism of MnO_2_, while most studies focused on various solid‐state analytical techniques, such as X‐ray diffraction (XRD), X‐ray photoelectron spectroscopy (XPS), and X‐ray absorption fine structure (XAFS). Only inductively coupled plasma (ICP) spectrometry, one of solution‐based analyses, was previously used to estimate the amount of Mn^2+^ dissolved in electrolytes.^[^ ^17^, ^32^ ^]^

Herein, we directly proved the reversible dissolution/deposition mechanism of Mn^2+^/Mn^4+^ through a 2e^−^ reaction at the MnO_2_ cathode for mild‐acid Zn‐MnO_2_ batteries. Solution‐based analyses, including ex situ electron spin resonance (ESR) spectroscopy and the designed electrochemical analysis operated with the two working electrodes system, were performed to demonstrate the reaction mechanism of MnO_2_. The initial cycle was remarkably irreversible. During the initial discharge, solid MnO_2_ (Mn^4+^) was reduced into Mn^2+^ (aq) through the one‐step two‐phase reaction. Soluble Mn^2+^ was then deposited on the cathode surface in the form of the mixture of the poorly crystalline Zn‐containing MnO_2_ compounds during charge through the two‐step two‐phase reactions. However, the subsequent cycles were reversible between the poorly crystalline Zn‐containing MnO_2_ and the dissolved Mn^2+^ ions. During cycling, the dissolved Mn^2+^ ions were diffused out from the cathode surface to the bulk electrolyte. As a result, the loss of electrochemically active Mn^2+^ ions increased gradually during cycling, leading to capacity fading. In this regard, a porous carbon interlayer was introduced to entrap the dissolved Mn^2+^ ions during cycling, resulting in the excellent electrochemical performance of pouch‐type Zn‐MnO_2_ batteries (dimension: 5 × 6 cm^2^ and nominal capacity: 70 mAh), such as negligible capacity fading over 100 cycles. We also fabricated prototype 20 Ah‐scale Zn‐MnO_2_ cells (25 × 30 cm^2^), showing a promising specific energy of ≈70 Wh kg^−1^.

## 2 Irreversible Phase Transitions for the Initial Cycle

**Figure 1:**
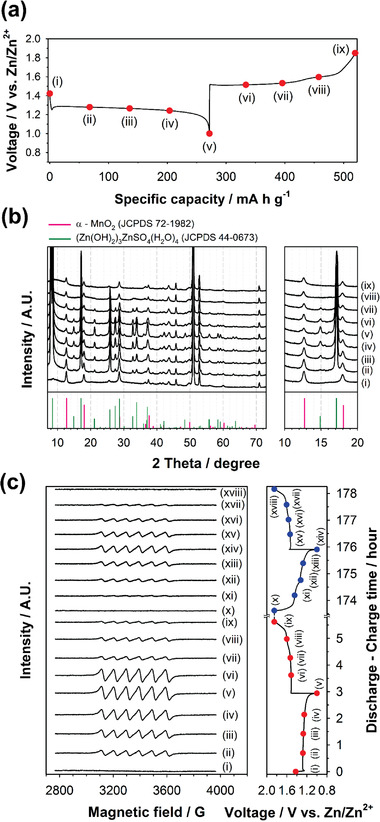
a) Voltage profile of mild‐acid Zn‐MnO_2_ cells at a specific current of 92.5 mA g^−1^ for the initial cycle. b) Ex situ XRD patterns of the *α*‐MnO_2_ cathodes retrieved at various discharge/charge states indicated in the voltage profile of (a). c) Ex situ ESR spectra of the electrolytes retrieved at various discharge/charge states indicated in the voltage profile of (c). A ZnSO_4_ aqueous solution (2 mol kg^−1^) containing no MnSO_4_ was used as the electrolyte.

We prepared *α*‐MnO_2_ nanorods as a cathode material using hydrothermal synthesis at 180 °C for 14 h.^[^ ^36^, ^37^ ^]^ No impurity phases were observed in the powder XRD pattern of *α*‐MnO_2_ nanorods (Figure S1a, Supporting Information). *α*‐MnO_2_ nanorods were ≈3 µm in length and ≈100 nm in thickness, as shown in the field emission scanning electron microscopy (FE‐SEM) image of *α*‐MnO_2_ (Figure S1b, Supporting Information). Zn metal foil and ZnSO_4_ aqueous solution (2 mol kg^−1^) were used as anode and electrolyte, respectively. **Figure** **1**a shows the voltage profile of mild acid Zn‐MnO_2_ cells for the initial cycle. Only one plateau was clearly observed at ≈1.28 V during the discharge, whereas the subsequent charge showed two plateaus at ≈1.51 and ≈1.60 V. This suggests that the discharge mechanism is different from the charge mechanism. We performed ex situ XRD and ESR analyses to demonstrate the irreversible reaction mechanism of the initial cycle. Figure 1b shows the ex situ XRD patterns of the *α*‐MnO_2_ electrodes retrieved at various discharge/charge states indicated in the voltage profile of Figure 1a. The XRD peak intensity of *α*‐MnO_2_ decreased gradually and the peak position remained unchanged during discharge. This indicates that *α*‐MnO_2_ was converted into another phase during discharge. At the same time, zinc hydroxy sulfate (ZHS), Zn_4_SO_4_(OH)_6_·4H_2_O, was newly appeared, and the intensity of ZHS increased and decreased gradually during discharge and charge, respectively. ZHS is known to form through a chemical precipitation from Zn^2+^, SO_4_^2−^, and OH^−^.^[^ ^38^, ^39^ ^]^ Considering the charge balance of the electrolyte, the formation of ZHS should be driven by the consumption of H^+^ as much as the equivalent amount of OH^−^ in ZHS. H^+^ ions were consumed through an electrochemical reaction with MnO_2_ during discharge.

For example, i) MnO_2_ + H^+^ + e^−^ → MnOOH, and/or ii) MnO_2_ + 2H^+^ + 2e^−^ → Mn^2+^ + 2OH^−^. However, no MnOOH peaks were observed during discharge, although the formation of MnOOH as a discharge product was reported in the previous literatures.^[^ ^16^, ^17^, ^29^ ^]^ This suggests that, during discharge, i) poorly crystalline MnOOH formed, ii) 2MnOOH was decomposed into Mn^2+^, 2OH^−^, and MnO_2_ via the disproportionation reaction of Mn^3+^, or iii) MnO_2_ was directly converted into Mn^2+^ and 2OH^−^ via a 2e^−^ reaction.

Moreover, the XRD peaks of *α*‐MnO_2_ were still intense even after full discharge, implying that a substantial amount of *α*‐MnO_2_ remained unreacted even after full discharge. In other words, despite the fact that the cell delivered the discharge capacity of 272 mA h g^−1^, which is ≈90% of the theoretical specific capacity (308 mA h g^−1^) of Mn^3+^/Mn^4+^ (MnO_2_ + H^+^ + e^−^ → MnOOH), a substantial amount of *α*‐MnO_2_ remained unreacted even after full discharge. This implies that the oxidation state of the discharge product was Mn^2+^ rather than Mn^3+^ after full discharge. In addition, no new XRD peaks appeared during charge and the peak intensity of *α*‐MnO_2_ was not increased and remained unchanged. This suggests that poorly crystalline or amorphous manganese oxides were formed during charge rather than crystalline *α*‐MnO_2_. This implies that the irreversible phase transitions of *α*‐MnO_2_ occurred during the initial cycle.

Figure 1c shows the ex situ ESR spectra of the electrolytes retrieved at various discharge/charge states indicated in the voltage profile of Figure 1c. Coin cells were disassembled after reaching each designated discharge/charge states. All parts of the disassembled coin cells, including the separator, were immersed in deionized water. The ESR spectra of the electrolyte solutions were then obtained after removing all solid parts of the coin cells. As the cell was discharged, a new ESR signal was appeared at a g‐value of 2. This ESR signal coincides with that of the MnSO_4_ (Mn^2+^) reference aqueous solution (Figure S2, Supporting Information), implying that the discharge product of MnO_2_ existed in the form of Mn^2+^ (aq) dissolved in the electrolyte.^[^ ^40^ ^]^ In contrast to Mn^2+^, no Mn^3+^ signals were observed in the ESR spectrum of the saturated aqueous solution of Mn(CH_3_COO)_3_ with 2 mol kg^−1^ ZnSO_4_ (Figure S3, Supporting Information). This is attributed to Kramer's theorem that the hyperfine lines for a *d* ^4^ system of a high‐spin Mn^3+^ are very weak compared to Mn^2+^ with *S* = 5/2.^[^ ^40^ ^]^ Moreover, the intensity of the Mn^2+^ signals increased and decreased gradually during discharge and charge, respectively, for the initial two cycles (Figure 1c). This reveals that the amount of Mn^2+^ in the electrolyte increased and decreased reversibly during discharge and charge, respectively.

**Figure 2:**
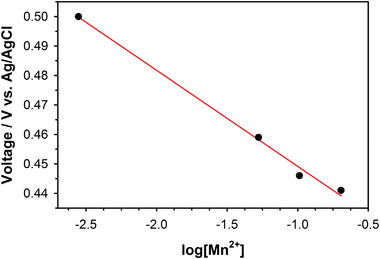
Correlation between the equilibrium potential of the MnO_2_ cathode and log [Mn^2+^] of the electrolyte.

To clarify the origin of the Mn^2+^ formation during discharge, we examined the correlation between the equilibrium potential of the MnO_2_ cathode and the Mn^2+^ concentration of the electrolyte. A beaker‐type cell was assembled with three electrodes, such as the *α*‐MnO_2_ working electrode, Zn metal counter electrode, and Ag/AgCl reference electrode. The *α*‐MnO_2_ electrode was discharged until the DOD was 20%, followed by measuring the open‐circuit potential of the *α*‐MnO_2_ electrode after resting for two hours to reach an equilibrium state. Then, various amounts of MnSO_4_ were added to the electrolyte solution to increase the Mn^2+^ concentration of the electrolyte in the concentration range between 0.003 and 0.203 mol kg^−1^. We also measured the open‐circuit potential of the *α*‐MnO_2_ electrode at various Mn^2+^ concentrations after each resting for two hours. **Figure** **2** shows the correlation between the equilibrium potential of the MnO_2_ cathode and the Mn^2+^ concentration of the electrolyte in the form of logarithm. The equilibrium potential of the MnO_2_ cathode was linearly proportional to log [Mn^2+^] of the electrolyte. This reveals that the electrochemically active species was Mn^2+^ rather than Mn^3+^. If Mn^3+^ was electrochemically active during discharge, the equilibrium potential of the MnO_2_ cathode should remained unchanged regardless of the concentration of Mn^2+^ in the electrolyte. This is because the activities of solid MnO_2_ and MnOOH are unity, as shown in the Nernst Equation (1).

(1) $$E=E^{0}-\frac{\mathrm{RT}}{\mathrm{zF}}\ln\frac{a_{\mathrm{MnOOH}}}{a_{\mathrm{Mn}O_{2}}}=E^{0}\;$$

However, if Mn^2+^ was electrochemically active during discharge, the equilibrium potential of the MnO_2_ cathode should decrease with increasing the concentration of Mn^2+^ in the electrolyte, as shown in the Nernst Equation (2).

(2) $$E=E^{0^{\prime }}-\frac{\mathrm{RT}}{\mathrm{zF}}\ln\frac{C_{{\mathrm{Mn}}^{2+}}}{C_{\mathrm{Mn}O_{2}}}=E^{0^{\prime }}-\frac{\mathrm{RT}}{\mathrm{zF}}\ln C_{{\mathrm{Mn}}^{2+}}$$

Moreover, from the fitting between the experimental data and the Nernst Equation (2), we found that *z* in the Nernst equation was ≈2. Therefore, this correlation implies that MnO_2_ was converted to Mn^2+^ through a 2e^−^ reaction during discharge.

**Figure 3:**
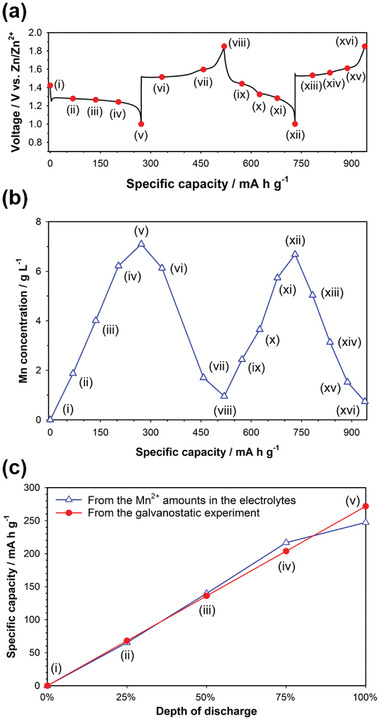
a) Voltage profile of the Zn‐MnO_2_ cell at a specific current of 92.5 mA g^−1^ for the initial two cycles. b) The amount of Mn^2+^ dissolved in the electrolyte of Zn‐MnO_2_ cells retrieved at various discharge/charge states indicated in the voltage profile of (a). A ZnSO_4_ aqueous solution (2 mol kg^−1^) containing no MnSO_4_ was used as the electrolyte. c) Comparison of the reversible capacity of MnO_2_ measured from the galvanostatic experiment with the capacity estimated from the amount of Mn^2+^ in the electrolyte.

We also performed ex situ inductively coupled plasma – atomic emission spectrometer (ICP‐AES) analysis to rigorously quantify the amounts of Mn^2+^ dissolved in the electrolyte at various discharge/charge states indicated in the voltage profile of **Figure** **3**a. In order to rigorously measure the amount of Mn^2+^ in the electrolyte, all parts of the coin cells were immersed in deionized water after disassembling the coin cell. We then measured the amount of Mn^2+^ in the solution using ICP‐AES after removing all solid parts of the coin cells, which include electrodes and a separator, using a centrifuge. Figure 3b shows that the amount of the dissolved Mn^2+^ increased and decreased gradually during cycling, which is consistent with the ex situ ESR analysis (Figure 3c). Moreover, we compared the reversible capacity of MnO_2_ measured from the galvanostatic experiment with the capacity estimated from the amount of Mn^2+^ in the electrolyte. The reaction of Mn^4+^ (in MnO_2_ (s)) + 2e^−^ ↔ Mn^2+^ (in the electrolyte) was considered to calculate the specific capacity estimated from the amount of Mn^2+^ in the electrolyte. It is remarkable that the reversible capacities estimated from the amounts of Mn^2+^ in the electrolytes were almost the same as the values measured from the galvanostatic experiment, as shown in Figure 3c. This implies that the discharge products of MnO_2_ existed only in the form of Mn^2+^ in the electrolyte during cycling. Note that the formation of Mn^2+^ (aq) was not due to the dissolution of MnO_2_, MnOOH, or the other manganese oxide compounds because their solubility in mild acidic aqueous solutions is negligible compared to the amount of Mn^2+^ in the electrolyte measured during discharge and charge (Figure S4, Supporting Information). Therefore, we suggest the discharge mechanism of the *α*‐MnO_2_ cathode for mild acid Zn‐MnO_2_ batteries as follows.

(3) $$\alpha -\mathrm{Mn}O_{2}\left(s\right)+2e^{-}+2H^{+}\left(\mathrm{aq}\right)\to Mn^{2+}\left(\mathrm{aq}\right)+2OH^{-}\left(\mathrm{aq}\right)$$

The 2e^−^ reaction of the MnO_2_ cathode between Mn^2+^ and Mn^4+^ were also supported by the discharge profiles of Zn‐MnO_2_ cells at various current densities (Figure S5, Supporting Information). The *α*‐MnO_2_ electrode delivered the discharge capacity of 371 mA h g^−1^ at a specific current of 7.7 mA g^−1^, which exceeds the theoretical capacity of *α*‐MnO_2_ for a 1e^−^ reaction between Mn^4+^ and Mn^3+^ (308 mA h g^−1^). This reveals that MnO_2_ was transformed to Mn^2+^ through a 2e^−^ reaction during discharge.

**Figure 4:**
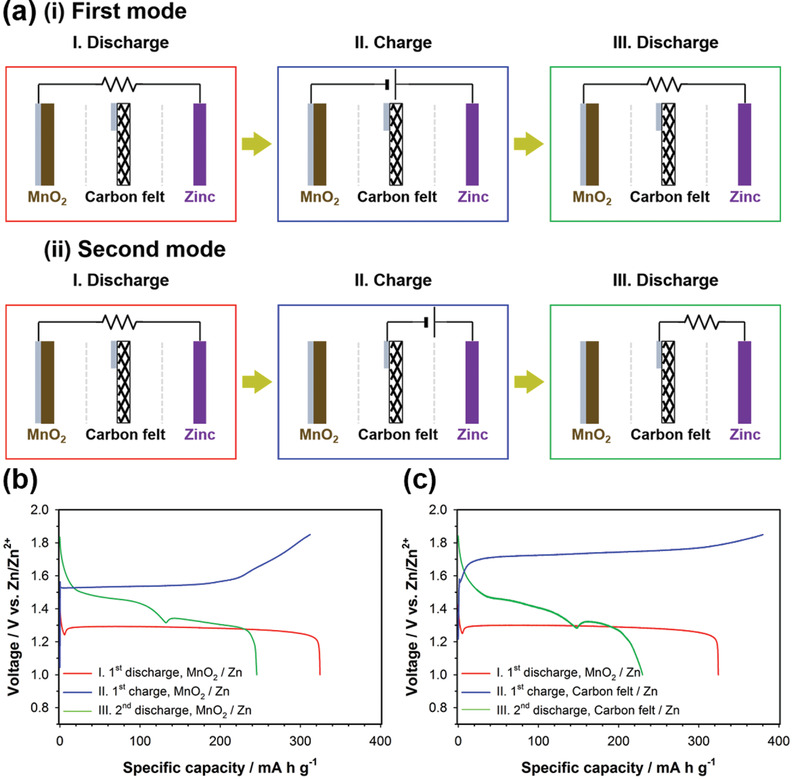
a) Schematic illustration for the discharge/charge modes of the two‐working electrodes system. One working electrode was the *α*‐MnO_2_ electrode. The other working electrode was a porous carbon felt, which was located between *α*‐MnO_2_ electrode and Zn metal electrode. A ZnSO_4_ aqueous solution (2 mol kg^−1^) containing no MnSO_4_ was used as the electrolyte. Voltage profiles of b) the first mode and c) the second mode in (a) at a constant current of 15.4 mA g^−1^.

To further support the reaction mechanism of *α*‐MnO_2_ into soluble Mn^2+^, we carried out the designed electrochemical experiments in which the cell was assembled with two working electrodes and one counter electrode (Zn metal foil), as shown in **Figure** **4**a. One working electrode was the *α*‐MnO_2_ electrode. The other working electrode was a porous carbon felt, which was located between *α*‐MnO_2_ electrode and Zn metal electrode. Three electrodes were electrically separated by insulating porous glass fiber membrane separators. Detailed cell components and cell assembly are displayed in Figure S6 in the Supporting Information. In the first mode, we connected the *α*‐MnO_2_ working electrode and the Zn metal counter electrode, and the cell, which was not connected with the carbon felt electrode, was cycled in the voltage range of 1.0–1.85 V (vs Zn/Zn^2+^) at a constant current of 15.4 mA g^−1^ (Figure 4ai). Ions as charge carriers can penetrate through the carbon felt electrode because the carbon felt is macroporous. The cell delivered ≈324 and 246 mA h g^−1^ of discharge capacity for the first and second cycles, respectively (Figure 4b). In the second mode, after the initial discharge between the *α*‐MnO_2_ working electrode and the Zn metal counter electrode, we disconnected the cell, and then, connected the carbon felt working electrode and the Zn metal counter electrode (Figure 4aii). The reconnected cell consisting of the carbon felt and the Zn metal electrodes was charged and then discharged in the voltage range of 1.0–1.85 V (vs Zn/Zn^2+^) at a constant current of 15.4 mA g^−1^. The discharge capacity of the carbon felt electrode in the reconnected cell was ≈230 mA h g^−1^ (Figure 4c), which is ≈93% of the second discharge capacity (246 mA h g^−1^) obtained from the first mode. This reveals that solid *α*‐MnO_2_ was reduced into only soluble Mn^2+^ during the initial discharge and almost all the dissolved Mn^2+^ ions were then deposited on the carbon felt surface during the subsequent charge. This implies that the discharge product was present only in the form of the dissolved Mn^2+^ ions. This is consistent with the ex situ ICP‐AES analysis of the electrolyte (Figure 3c).

**Figure 5:**
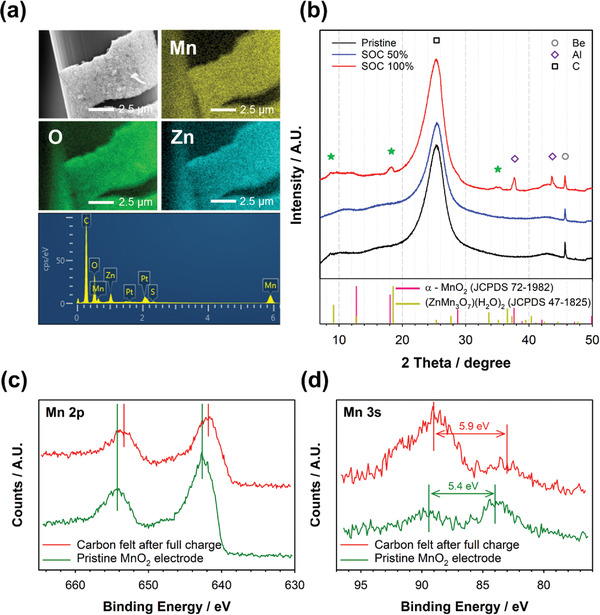
a) Ex situ SEM and EDS mapping images and the corresponding EDS spectrum of the carbon felt electrode after full charge in the second mode of Figure 4. b) Ex situ XRD patterns of the carbon felt electrodes retrieved at various SOC states. c) Ex situ Mn 2p and d) Mn 3s XPS spectra of the carbon felt electrodes (pristine vs after full charge).

In addition, the charge products deposited on the carbon felt surface were clarified using ex situ SEM‐EDS, XRD, and XPS analyses. **Figure** **5**a and Figure S7 (Supporting Information) show the SEM and corresponding EDS mapping images of the carbon felt surface retrieved at various state‐of‐charge (SOC) states. Urchin‐like deposits were observed as charge products, and the amount of the deposits increased gradually during charge (Figure S7, Supporting Information). The charge products were composed of Zn, Mn, and O elements. S peaks were negligible in the EDS spectrum of the carbon felt, while Zn peaks were clearly observed (Figure 5a). This implies that Zn on the carbon felt surface was not originated from ZHS because ZHS includes not only Zn but also S, as shown in the SEM and EDS mapping images of ZHS (Figure S8, Supporting Information). Figure 5b shows the XRD pattern of the carbon felt electrodes retrieved at the SOC of 50% and 100%. No new peaks were appeared at the SOC of 50%, implying that the charge products at the SOC of 50% were amorphous or poorly crystalline. At the SOC of 100%, however, we observed small XRD peaks at ≈18°, 29°, and 35°, which correspond to ZnMn_3_O_7_·2H_2_O. This suggests that the zinc manganese oxide with a 3 × 4 microtunnel structure formed after full charge, which is different from the pristine *α*‐MnO_2_.^[^ ^24^, ^30^ ^]^ We also compared the Mn 2p and Mn 3s XPS spectra of the pristine *α*‐MnO_2_ and the carbon felt surface obtained after full charge. The Mn 2p peaks of the pristine *α*‐MnO_2_ were located at higher binding energies than those of the charge products on the carbon felt surface (Figure 5c). The magnitude of Mn 3s peak splitting of the charge products on the carbon felt surface also increased compared to the pristine *α*‐MnO_2_ (Figure 5d). The magnitude of Mn 3s peak splitting is dependent on the Mn oxidation state because of the coupling of non‐ionized 3s electron with 3d valence‐band electrons. These XPS results reveal that the Mn average oxidation state of the charge products on the carbon felt surface was lower than that of the pristine *α*‐MnO_2_.^[^ ^41^ ^]^ This indicates that the average oxidation state of Mn ions was not fully restored into that of the pristine *α*‐MnO_2_ after full charge.

**Figure 6:**
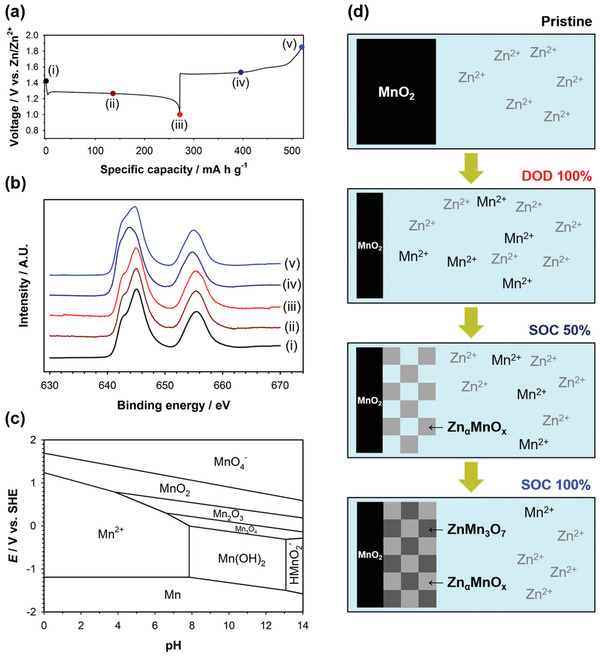
a) Voltage profile of the Zn‐MnO_2_ cell at a specific current of 92.5 mA g^−1^ for the first cycle. b) Ex situ soft XAS spectra (TEY mode) at the Mn L_2,3_ absorption edges for the *α*‐MnO_2_ electrodes retrieved at various discharge/charge states indicated in (a). A ZnSO_4_ aqueous solution (2 mol kg^−1^) containing no MnSO_4_ was used as the electrolyte. c) Eh‐pH diagram of the Mn‐H_2_O system ([Mn^2+^] = 0.38 m) at 25 °C. d) Schematic illustration for the discharge and charge mechanism of the MnO_2_ cathode for the first cycle.

We also performed ex situ soft X‐ray absorption spectroscopy (sXAS) and X‐ray absorption near‐edge structure (XANES) spectroscopy using synchrotron radiation to elucidate the discharge and charge mechanism of MnO_2_ in more detail (**Figure** **6**; Figure S9, Supporting Information). Coin cells were disassembled after reaching each designated discharge/charge states. The MnO_2_ electrodes were then washed with deionized water, followed by drying at room temperature. Figure 6b shows the surface sensitive sXAS spectra (TEY mode) at the Mn L_2,3_ absorption edges for the *α*‐MnO_2_ electrodes retrieved at various discharge/charge states indicated in Figure 6a. The Mn L edge spectra remained unchanged during discharge, implying that the oxidation state of MnO_2_ electrode was not changed during discharge. This supports our suggested discharge mechanism: i) solid MnO_2_ was transformed into soluble Mn^2+^ during discharge and ii) some *α*‐MnO_2_ remained unreacted even after full discharge. However, we observed changes in the oxidation state of Mn during charge. It is remarkable that the Mn L edge sXAS spectra shift to the lower energies at the SOC of 50% and then back to the higher energies at the SOC of 100%, indicating that the average oxidation state of Mn ions decreased and then increased again during charge.^[^ ^42^, ^43^, ^44^ ^]^ Figure S9 in the Supporting Information shows the ex situ Mn K‐edge XANES spectra of MnO_2_ electrodes retrieved at various discharge/charge states. The oxidation states of Mn were calibrated using the linear correlation between oxidation states and XANES edge positions of the reference standards of Mn^II^O, Mn^III^OOH, and Mn^IV^O_2_. The oxidation states of Mn remained almost unchanged during discharge. During charge, however, the average oxidation state of Mn decreased and then increased again. This behavior is consistent with ex situ sXAS (Figure 6b). Therefore, the sXAS and XANES results suggest that the dissolved Mn^2+^ ions were deposited in two steps during charge. In the first step, Mn^2+^ ions were deposited on the cathode surface in the form of amorphous Zn‐containing MnO*_x_*, Zn*_*α*_*MnO*_x_*, in which the Mn oxidation state was lower than 4 (*Z* < 4 for Mn^z+^). Then, in the second step, Mn^2+^ ions were deposited in the form of another Zn‐containing MnO_2_, such as ZnMn_3_O_7_·2H_2_O, in which *Z* = 4 for Mn*^z^* ^+^. This two‐step charge mechanism gave rise to two plateaus in the charge voltage profile. The two‐step deposition of Mn^2+^ was due to a decrease in the pH of the electrolyte during charge. OH^−^ ions were consumed for the formation of manganese oxides during charge. As a result, the pH of the electrolyte decreased during charge.^[^ ^39^ ^]^ Figure 6c shows the Pourbaix diagram of the Mn‐H_2_O system at 25 °C.^[^ ^45^ ^]^ The oxidation state of predominant solid Mn compounds in the mild acidic region increased (Mn_2_O_3_ → MnO_2_) with (i) decreasing the pH of the solution as well as (ii) increasing the potential of the cell. For this reason, Mn^2+^ ions were first deposited in the form of Zn*_*α*_*MnO*_x_* (*Z* < 4 for Mn^z+^), and then deposited in the form of ZnMn_3_O_7_·2H_2_O (*Z* = 4 for Mn^z+^). Moreover, the Mn L edge sXAS peaks of the MnO_2_ electrode obtained after full charge were located at lower energies than those of the pristine *α*‐MnO_2_ electrode. This indicates that the average oxidation state of Mn ions was not fully restored into that of the pristine *α*‐MnO_2_ (Mn^4+^) after full charge, which is consistent with the XPS results in Figure 5. This supports the formation of the mixture of Zn*_*α*_*MnO*_x_* (*Z* < 4 for Mn^z+^) and ZnMn_3_O_7_·2H_2_O (*Z* = 4 for Mn^z+^) after full charge. Taking the ex situ sXAS analysis into account, the charge mechanism of the *α*‐MnO_2_ cathode for mild acid Zn‐MnO_2_ batteries is suggested as follows

In the first step

(4) $$\begin{array}{ccc} & & Mn^{2+}\left(\mathrm{aq}\right)+\alpha Zn^{2+}\left(\mathrm{aq}\right)+xOH^{-}+yH_{2}O\left(l\right)\to \\ & & Zn_{\alpha }\mathrm{Mn}O_{x}yH_{2}O\left(s\right)+xH^{+}\left(\mathrm{aq}\right)+2\left(x-\alpha -1\right)e^{-} \end{array}$$

In the second step

(5) $$\begin{array}{ccc} & & 3Mn^{2+}\left(\mathrm{aq}\right)+Zn^{2+}\left(\mathrm{aq}\right)+7OH^{-}+2H_{2}O\left(l\right)\to \\ & & \mathrm{ZnM}n_{3}O_{7}2H_{2}O\left(s\right)+7H^{+}\left(\mathrm{aq}\right)+6e^{-} \end{array}$$

Figure 6d schematically summarizes the discharge and charge mechanism of the MnO_2_ cathode for the first cycle.

We also performed ex situ TEM and EDS analyses to support the charge mechanism of mild acid Zn‐MnO_2_ batteries. Figures S10 and S11 in the Supporting Information show ex situ TEM and EDS mapping images and the corresponding EDS spectra at the SOC of 50% and 100%, respectively. Urchin‐like precipitates were observed as charge products at both SOC of 50% and 100%, which is consistent with ex situ SEM images of the charge products (Figure S7, Supporting Information). In addition, the EDS peak intensity ratio of Zn to Mn for the charged product at the SOC of 100% was higher than that at the SOC of 50% (Figures S10e and S11e, Supporting Information). This reveals that the Zn/Mn atomic ratio of the charged products increased gradually during charge, rather than decreased due to of the de‐insertion of Zn^2+^ during charge. If Zn_*α*_MnO_x_ was further oxidized into ZnMn_3_O_7_·2H_2_O through the de‐insertion of Zn^2+^ during charge, the Zn/Mn atomic ratio of the charged products should decrease with increasing SOC. Therefore, this implies that Mn^2+^ was stepwise precipitated to Zn_*α*_MnO_x_ and then ZnMn_3_O_7_·2H_2_O. In other words, Zn_*α*_MnO_x_ and ZnMn_3_O_7_·2H_2_O were generated directly and sequentially from Mn^2+^ ions dissolved in the electrolyte during charge.

**Figure 7:**
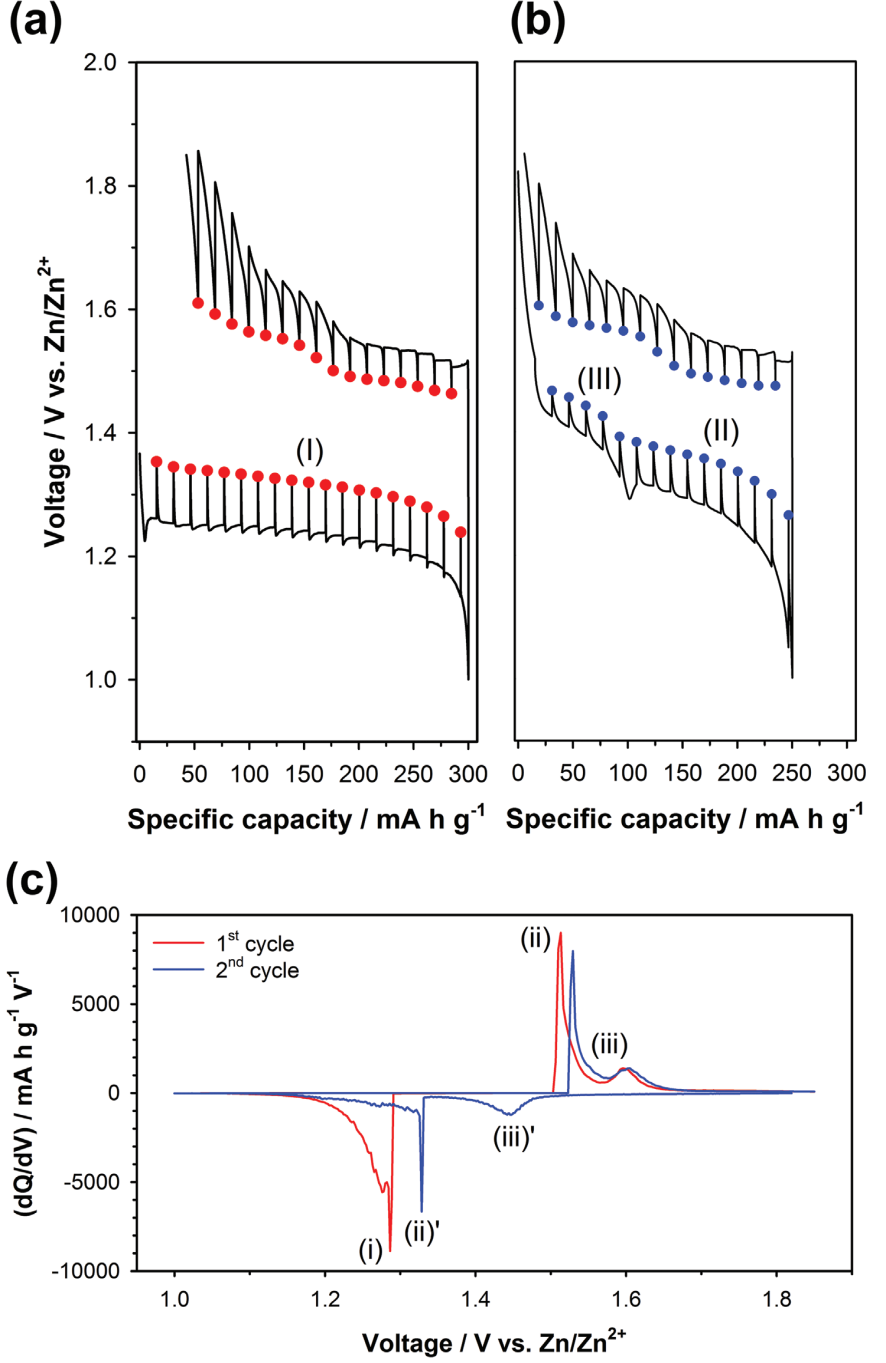
QOCV profiles of the Zn‐MnO_2_ cell for a) the first cycle and b) the second cycle. c) dQ/dV plots of the Zn‐MnO_2_ cell for the first and second cycles. A ZnSO_4_ aqueous solution (2 mol kg^−1^) containing no MnSO_4_ was used as the electrolyte.

**Figure** **7**a,b compares the quasi‐open‐circuit voltage (QOCV) profiles of mild acid Zn‐MnO_2_ cells for the first and second cycles. The QOCV profiles were measured using the galvanostatic intermittent titration technique (GITT). The first and second cycles showed nearly the same quasi‐equilibrium potentials during charge. In contrast to charge, the quasi‐equilibrium potentials of the first discharge were different from those of the second discharge. Only one plateau (I) was observed in the first discharge, whereas the second discharge showed two plateaus (II) and (III). This is attributed to that the charge product, such as the mixture of Zn_*α*_MnO_x_ and ZnMn_3_O_7_·2H_2_O, obtained after the first cycle showed a different discharge mechanism from the pristine *α*‐MnO_2_. Figure 7c shows the dQ/dV plots of mild acid Zn‐MnO_2_ cells for the first and second cycles. In consistent with the QOCV profiles, the dQ/dV plot of the first cycle was asymmetric, where one reduction peak (i) and two oxidation peaks (ii) and (iii) were observed. However, mild acid Zn‐MnO_2_ cells showed the symmetric dQ/dV plots for the subsequent cycles. Two reduction peaks (ii)’ and (iii)’ were also observed during discharge for the second cycle. Each reduction of peak (ii)’ and (iii)’ is paired with each oxidation of peak (ii) and (iii), respectively. This reveals that both charge and discharge proceeded each through two‐step two‐phase reactions after the first cycle. These results suggest that *α*‐MnO_2_ was converted into Mn^2+^ during discharge for the first cycle, which corresponds to peak (i). Mn^2+^ was irreversibly transformed to the mixture of Zn_*α*_MnO_x_ and ZnMn_3_O_7_·2H_2_O during charge, which correspond to peaks (ii) and (iii), respectively. Then, each Zn_*α*_MnO_x_ and ZnMn_3_O_7_·2H_2_O was reversibly transformed into Mn^2+^ during the subsequent cycles, which correspond to peak (ii)’ and (iii)’, respectively.

We also investigated the reaction mechanism of *β*‐MnO_2_ nanorods, the polymorph of *α*‐MnO_2_. Figure S12a,b in the Supporting Information compares the XRD pattern and SEM image of *β*‐MnO_2_ nanorods. Figure S12c,d in the Supporting Information shows the voltage profiles of *α*‐MnO_2_ and *β*‐MnO_2_ cathodes for the first and second cycles, respectively. Both *α*‐MnO_2_ and *β*‐MnO_2_ electrodes showed the almost same voltage profiles. Only one plateau was clearly observed during the initial discharge, whereas the subsequent charge and discharge showed two plateaus. This suggests that their reaction mechanisms are similar. Moreover, we performed ex situ ESR analysis to demonstrate the same dissolution/deposition (Mn^2+^/Mn^4+^) mechanism of *β*‐MnO_2_ as *α*‐MnO_2_. Figure S12e in the Supporting Information shows the ex situ ESR spectra of the *β*‐MnO_2_ electrolytes retrieved at various discharge/charge states indicated in the voltage profile of Figure S12e in the Supporting Information. The intensity of the Mn^2+^ signals increased and decreased gradually during discharge and charge, respectively, which is consistent with ex situ ESR spectra of *α*‐MnO_2_ (Figure 1c). This implies that *β*‐MnO_2_ showed the same reaction mechanism as *α*‐MnO_2_.

## 3 Capturing Dissolved Mn^2+^ Ions Using a Carbon Interlayer for Zn‐MnO_2_ Cells

**Figure 8:**
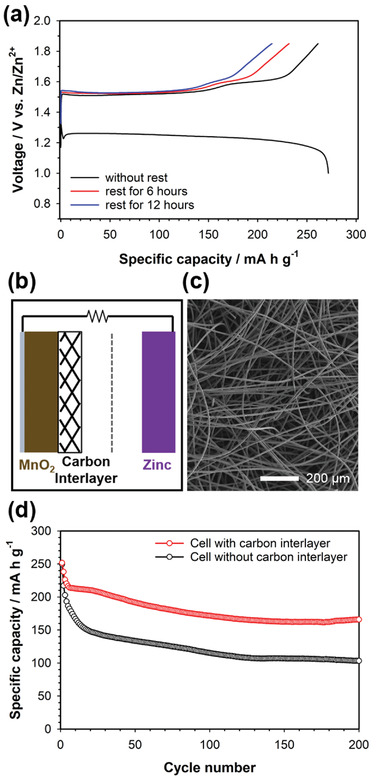
a) Voltage profiles of the Zn‐MnO_2_ cells for the first cycle with various rest times between discharge and charge. b) Schematic illustration for Zn‐MnO_2_ cells with the porous carbon interlayer. c) SEM image of the porous carbon felt interlayer. d) Cycle performances of Zn‐MnO_2_ coin cells with and without the carbon interlayer. A ZnSO_4_ aqueous solution (2 mol kg^−1^) with MnSO_4_ (0.1 mol kg^−1^) was used as the electrolyte.

**Figure 9:**
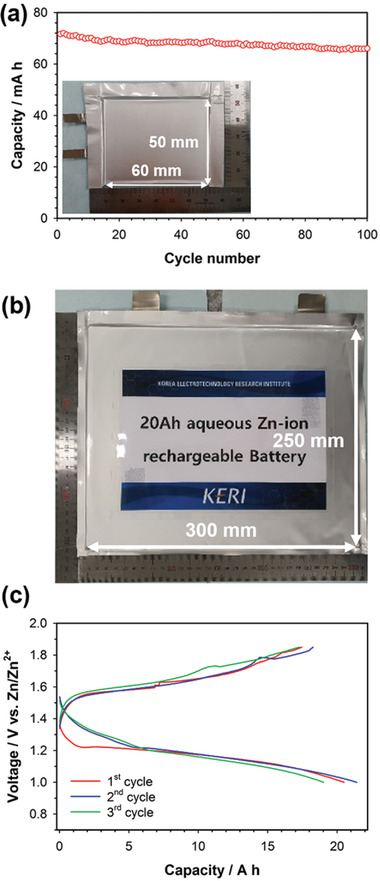
a) Cycle performance of the pouch‐type Zn‐MnO_2_ cell with the carbon interlayer (nominal capacity: ≈70 mAh, inset: the picture of the pouch‐type Zn‐MnO_2_ cell). b) Picture of the 20 Ah‐scale Zn‐MnO_2_ cell. c) Voltage profiles of the prototype 20 Ah‐scale Zn‐MnO_2_ cell. A ZnSO_4_ aqueous solution (2 mol kg^−1^) with MnSO_4_ (0.1 mol kg^−1^) was used as the electrolyte.

**Figure** **8**a compares the voltage profiles of mild acid Zn‐MnO_2_ cells with various rest times between discharge and charge. The charge capacity decreased gradually with increasing rest times. This suggests that the dissolved Mn^2+^ ions formed during discharge gradually diffused out from the electrode surface into the bulk electrolyte during the rest time. As a result, Mn^2+^ ions far from the electrode surface became electrochemically inactive. Therefore, the capacity fading of Zn‐MnO_2_ cells was due to the loss of electrochemically active Mn^2+^ ions during cycling. In this regard, we introduced a porous carbon felt interlayer on the MnO_2_ electrode surface to suppress the loss of Mn^2+^ ions (Figure 8b). Figure 8c shows the SEM image of the porous carbon felt interlayer with a thickness of ≈50 µm. We compared the cycle performances of mild acid Zn‐MnO_2_ coin cells with and without the carbon interlayer, as shown in Figure 8d. The Zn‐MnO_2_ cell with the carbon interlayer showed much more stable capacity retention over 200 cycles than did that without the carbon interlayer. The improved cycle performance of the carbon interlayer is attributed to that the carbon interlayer entrapped the dissolved Mn^2+^ ions diffused out to the bulk electrolyte during cycling.^[^ ^46^ ^]^ Moreover, we fabricated the pouch‐type Zn‐MnO_2_ cells with the carbon interlayer. Four sheets of the MnO_2_ cathode and five sheets of the Zn metal anode were stacked for the assembly of the pouch‐type full cells (5 × 6 cm^2^), which have a nominal capacity of ≈70 mAh. The Zn metal electrodes were prepared in the form of the composite electrode consisting of Zn metal powers, PVdF binder, and super P carbon in a weight ratio of 8:1:1. Zn metal powders were a few micrometers in size, as shown in the SEM image of Figure S13 in the Supporting Information. The pouch‐type Zn‐MnO_2_ cell with the carbon interlayer also showed excellent electrochemical performance, such as negligible capacity fading over 100 cycles (**Figure** **9**a). We also prepared the prototype 20 Ah‐scale Zn‐MnO_2_ cells (25 × 30 cm^2^), as shown in the picture of Figure 9b and the voltage profile of Figure 9c. 15 sheets of the MnO_2_ cathode and 16 sheets of the Zn metal anode were stacked for the assembly of the 20 Ah‐scale cells. The detailed specification of the 20 Ah‐scale full cell is presented in Table S1 in the Supporting Information. The 20 Ah‐scale cell showed the high specific energy of 70 Wh kg^−1^, which was calculated based on the total mass of the cell including pouch cell packaging. This validates that aqueous Zn‐MnO_2_ batteries are promising as a power source for large scale energy storage systems.

## 4 Conclusion

The reversible dissolution/deposition (Mn^2+^/Mn^4+^) mechanism of the MnO_2_ cathode for mild‐acid Zn‐MnO_2_ batteries was directly evidenced using solution‐based analyses, such as ex situ ESR spectroscopy and the designed electrochemical experiments operated with the two working electrodes system, in combination with solid‐state analytical techniques, including ex situ soft XAS, XANES, XRD, TEM, and XPS. We demonstrated that irreversible phase transitions occurred during the first cycle. Solid *α*‐MnO_2_ (Mn^4+^) was transformed into Mn^2+^ (aq) via the one‐step two‐phase reaction during the initial discharge. The discharge product existed only in the form of Mn^2+^ (aq) during discharge. Dissolved Mn^2+^ ions were then deposited on the cathode surface in the form of the mixture of the poorly crystalline Zn‐containing MnO_2_ compounds during charge through the two‐step two‐phase reactions. However, the subsequent cycles showed the reversible phase transitions between the poorly crystalline Zn‐containing MnO_2_ and the dissolved Mn^2+^ ions during discharge and charge.

In addition, we investigated the failure mechanism of mild‐acid Zn‐MnO_2_ batteries, in which the dissolved Mn^2+^ ions were diffused out to bulk electrolytes during cycling. This diffusion gave rise to the loss of electrochemically active Mn^2+^, resulting in poor cycle performance. In this regard, the porous carbon interlayer was introduced to entrap the dissolved Mn^2+^ ions during cycling. The carbon interlayer suppressed the loss of Mn^2+^, leading to the excellent electrochemical performance of pouch‐type Zn‐MnO_2_ full cells (dimension: 5 × 6 cm^2^ and nominal capacity: 70 mAh), such as negligible capacity fading over 100 cycles. We also fabricated prototype 20 Ah‐scale Zn‐MnO_2_ cells (25 × 30 cm^2^), showing a promising specific energy of ≈70 Wh kg^−1^ based on the total mass of the cell including pouch cell packaging. Our findings provide fundamental insights into strategies to improve the electrochemical performance of aqueous Zn‐MnO_2_ batteries.

## 5 Experimental Section

##### Material and Synthesis

*α*‐MnO_2_ nanorods were synthesized through a hydrothermal method. KMnO_4_ (3.75 g, 99%, Sigma‐Aldrich) and MnSO_4_·H_2_O (0.21 g, 99%, Sigma‐Aldrich) were dissolved in deionized water (240 mL), and the mixture was then loaded into a Teflon‐lined autoclave (300 mL) and heated at 180 °C for 14 hours. The obtained products were filtered, washed thoroughly using DI water, and dried at 80 °C for 12 hours. *β*‐MnO_2_ nanorods were also obtained using the same method as *α*‐MnO_2_ nanorods, but Mn(CH_3_COO)_2_·4H_2_O (7.59 g, 99.99%, Sigma‐Aldrich) and (NH_4_)_2_S_2_O_8_ (7.06 g, 97%, Sigma‐Aldrich) were used as precursors for *β*‐MnO_2_ nanorods.

##### Material Characterization

The XRD patterns of powders and electrodes were obtained using a Bruker D2 PHASER with Cu K*α* radiation (*λ* = 1.5418 Å) operated in the 2*θ* range of 7–80°. For XRD analysis, airtight sample holders equipped with a Be window were used. For ex situ analyses, cells were disassembled in air and electrode samples were rinsed with deionized water before the measurement. SEM and EDS were carried out on a field emission scanning electron microscope with accelerating voltage of 15 kV (JEOL FE‐SEM 7800F Prime). TEM analysis was carried out using a scanning transmission electron microscope (JEOL JEM‐2100F). ESR spectra were recorded at room temperature with a Bruker EMXmicro‐9.5/2.7 X‐band spectrometer with high‐sensitive cavity E4119001, which operates at X‐band frequencies (≈9.4 GHz). The field sweep was set from 0 to 2000 G and a sweep time was set to 120 s. Attenuation was set to 25 dB. The modulation amplitude was set to 1.0 G at a modulation frequency of 100 kHz. The sXAS spectra of the Mn K‐edge were obtained at the bending magnet beamline 10D – XAS KIST (Korea Institute of Science and Technology) of Pohang Accelerator Laboratory (PAL). The storage ring was operated with a ring current of 360 mA at 3.0 GeV in top‐up mode. sXAS spectra were collected in total electron yield (TEY) mode at a penetration depth of <10 nm and normalized to the incident photon flux at an energy resolution of 0.1 eV. XANES analysis was performed at the magnet beamline PLS‐II 7D XAFS of PAL. XANES spectra of Mn K‐edge were obtained using a Si(111) double‐crystal monochromator in transmission mode at an electron energy of 3 GeV and a current of 360 mA during top‐up mode operation. ICP‐AES was performed using OPTIMA 8300 atomic emission spectrometer (Perkin‐Elmer, USA) with argon plasma source.

##### Electrochemical Characterization of Coin Cells

The electrochemical performance of coin cells was evaluated using 2032‐type coin cells with a MnO_2_ electrode, a Zn metal foil (0.25 mm in thickness, 99.98%, Alfa Aesar), and a glass fiber separator. For the preparation of MnO_2_ electrodes, MnO_2_ was mixed with carbon black (Super P, TIMCAL) and polyvinylidene fluoride (PVdF, KF‐1100, Kureha) in a weight ratio of 6:2:2. The slurry was casted onto a current collector (SUS316L foil, 20 µm in thickness). The electrodes were dried at 100 °C for 10 min. For various ex situ analyses, ZnSO_4_ aqueous solutions (40 µL, 2 mol kg^−1^) containing no MnSO_4_ were used as electrolytes. For the evaluation of cycle performance of coin cells, a ZnSO_4_ aqueous solution (2 mol kg^−1^) containing MnSO_4_ (0.1 mol kg^−1^) was used as the electrolyte. For the assembly of the designed electrochemical cells with two working electrodes, *α*‐MnO_2_ electrode and porous carbon felt (TOYOBO XF30A Activated carbon felt) were used as the working electrodes. Galvanostatic cycling was performed in a voltage range of 1.0–1.85 V (vs Zn/Zn^2+^) at a specific current of 92.5 mA g^−1^ using a WBCS3000 battery cycler (WonATech, S. Korea) at 30 °C.

##### Equilibrium Potential Measurement Using a Beaker‐Type Cell

The equilibrium potential of the MnO_2_ cathode was measured at various Mn^2+^ concentration in the electrolyte using a beaker‐type cell that assembled with the *α*‐MnO_2_ working electrode, Zn metal counter electrode, and Ag/AgCl reference electrode. A ZnSO_4_ aqueous solution (100 mL, 2 mol kg^−1^) was used as an electrolyte. The *α*‐MnO_2_ electrode was discharged until the DOD was 20%, followed by measuring the open‐circuit potential of the *α*‐MnO_2_ electrode after resting for two hours to reach an equilibrium state. Various amounts of MnSO_4_ were also added to the electrolyte solution to change the Mn^2+^ concentration of the electrolyte in the concentration range of 0.003–0.203 m. The open‐circuit potential of the *α*‐MnO_2_ electrode was then measured after each resting for two hours.

##### Electrochemical Characterization of Pouch Cells

20 Ah‐scale Zn‐MnO_2_ cells (25 × 30 cm^2^) were stacked with 15 sheets of the MnO_2_ cathode and 16 sheets of the Zn metal anode. 70 mAh‐scale Zn‐MnO_2_ cells (5 × 6 cm^2^) were stacked with 4 sheets of the MnO_2_ cathode and 5 sheets of the Zn metal anode. Zn metal electrodes were prepared in the form of the composite electrode consisting of Zn metal powers, PVdF binder, and super P carbon in a weight ratio of 8:1:1. The cell performances of pouch‐type Zn‐MnO_2_ cells were evaluated in a voltage range of 1.0–1.85 V (vs Zn/Zn^2+^) at 20 mA and 2 A for 70 mAh and 20 Ah cells, respectively. The detailed specification of pouch cells is presented in Table S1 in the Supporting Information.

## Conflict of Interest

The authors declare no conflict of interest.

## Supporting information

Supporting Information
