# Supplementary material for: Direct Proof of the Reversible Dissolution/Deposition of Mn2+/Mn4+ for Mild‐Acid Zn‐MnO2 Batteries with Porous Carbon Interlayers
Source: Adv Sci (Weinh). 2021 Feb 1;8(6):2003714. doi: 10.1002/advs.202003714 (PMC7967064; doi:10.1002/advs.202003714)
Supplement: Supplementary file 1 — Supporting Information [file ADVS-8-2003714-s001.pdf]

© 2020 Wiley-VCH GmbH

ADVANCED SCIENCE
Open Access

Supporting Information

for *Adv. Sci.,* DOI: 10.1002/advs.202003714

Direct Proof of the Reversible Dissolution/Deposition of Mn^{2+}/Mn^{4+} for Mild-Acid Zn-MnO_2 Batteries with Porous Carbon Interlayers

Hyeonseok Moon, Kwang-Ho Ha, Yuwon Park, Jungho Lee, Mi-Sook Kwon, Jungwoo Lim, Min-Ho Lee, Dong-Hyun Kim, Jin H. Choi, Jeong-Hee Choi, and Kyu Tae Lee**

WILEY-VCH

Copyright WILEY-VCH Verlag GmbH & Co. KGaA, 69469 Weinheim, Germany, 2018.

Supporting Information

Direct Proof of the Reversible Dissolution/Deposition of Mn^{2+}/Mn^{4+} for Mild-Acid Zn-MnO_2 Batteries with Porous Carbon Interlayers

Hyeonseok Moon[†], Kwang-Ho Ha[†], Yuwon Park, Mi-Sook Kwon, Jungwoo Lim, Min-Ho Lee, Dong-Hyun Kim, Jin H. Choi, Jeong-Hee Choi and Kyu Tae Lee**

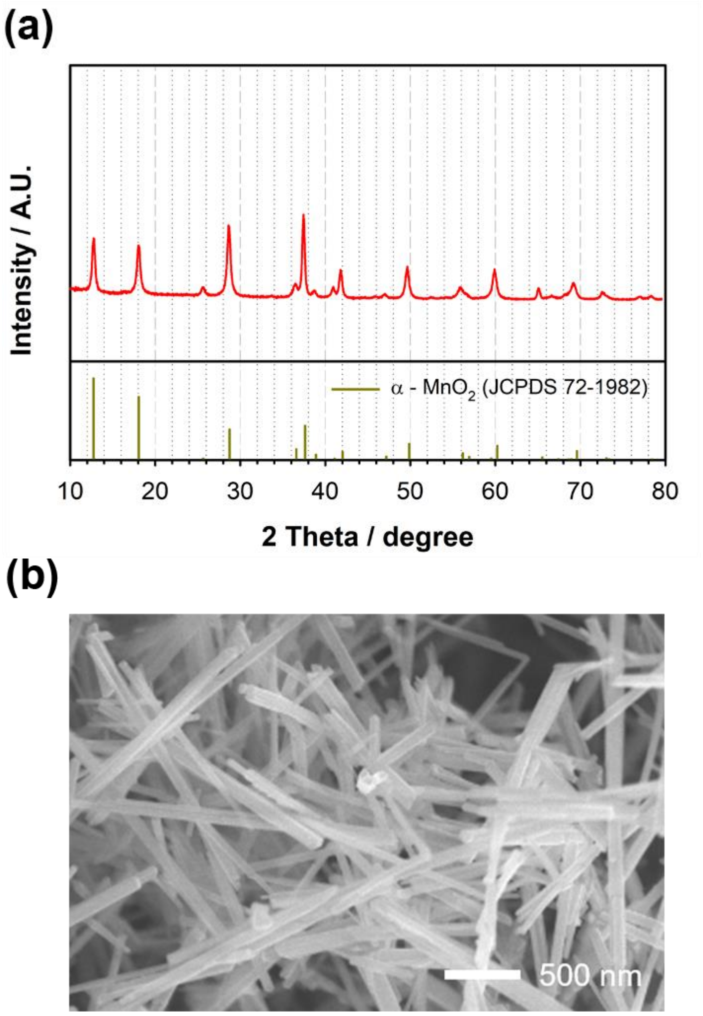

Figure S1. (a) XRD pattern and (b) FE-SEM image of α-MnO_2 nanorods.

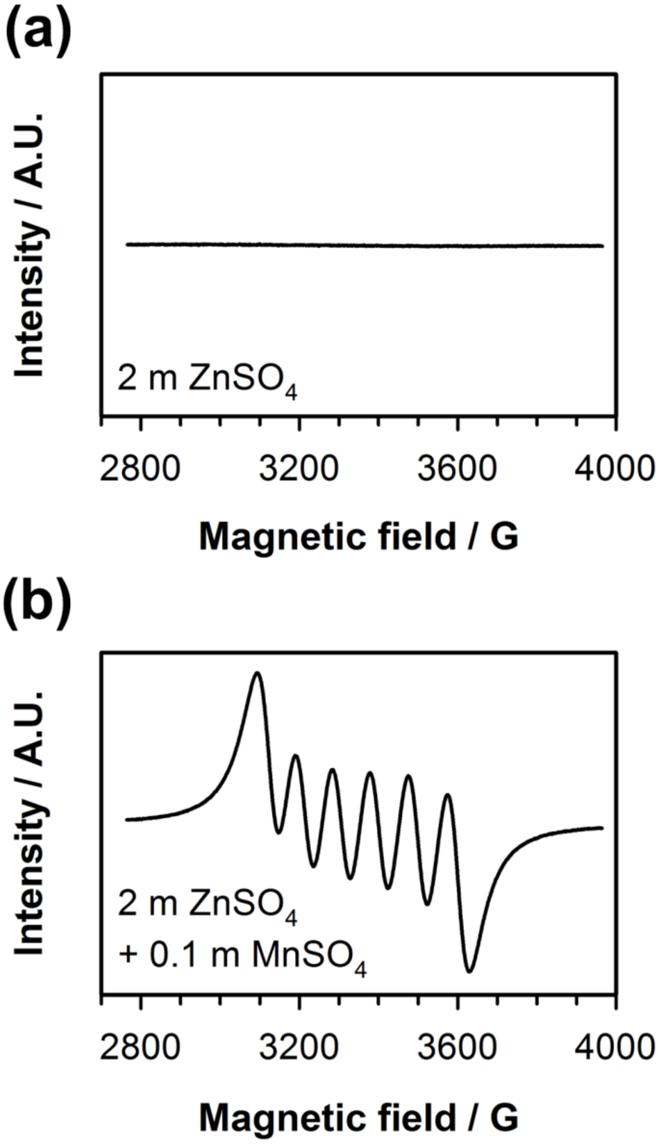

Figure S2. ESR spectra of aqueous (a) $ZnSO_4$ (2 mol kg^{-1}) and (b) $ZnSO_4$ (2 mol kg^{-1}) + $MnSO_4$ (0.1 mol kg^{-1}) electrolytes.

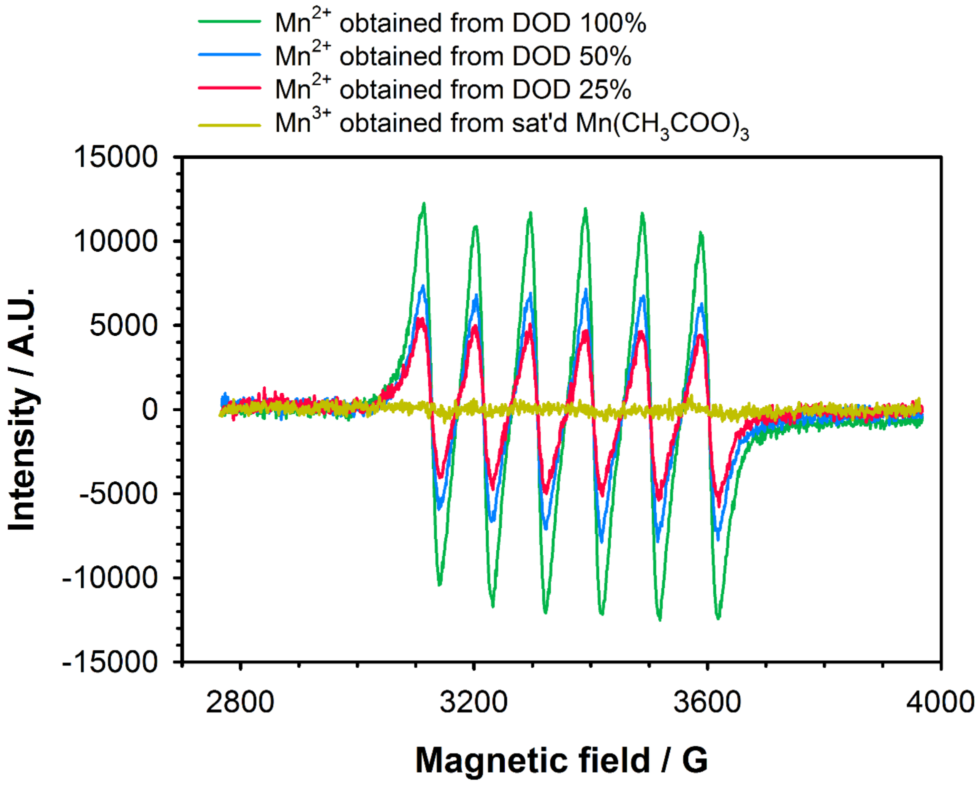

Figure S3. ESR spectra of the electrolytes retrieved at various DOD states of Zn-MnO_2 cells and the saturated aqueous solution of $Mn(CH_3COO)_3$ with $ZnSO_4$ (2 mol kg^{-1}).

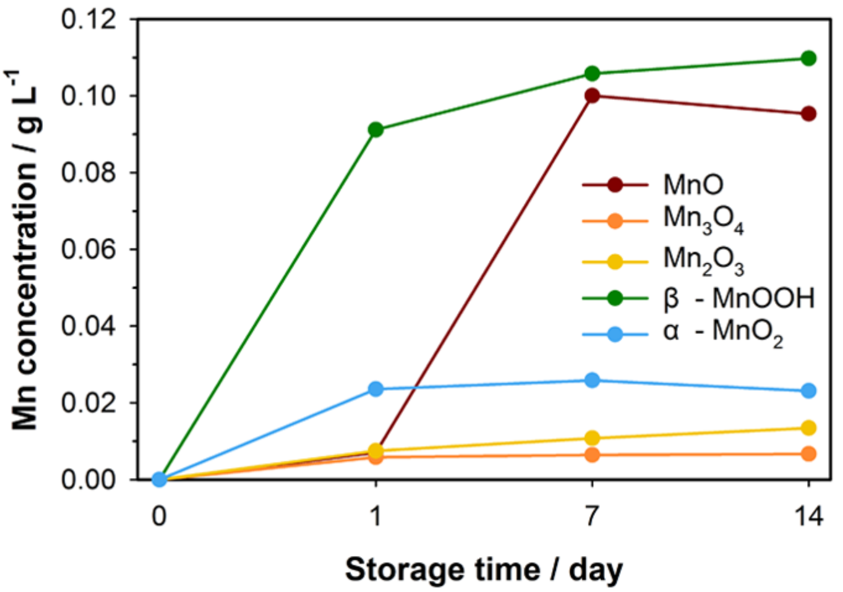

Figure S4. Solubility of various manganese oxide compounds in an aqueous $ZnSO_4$ solution (2 mol kg^{-1}) for various storage times.

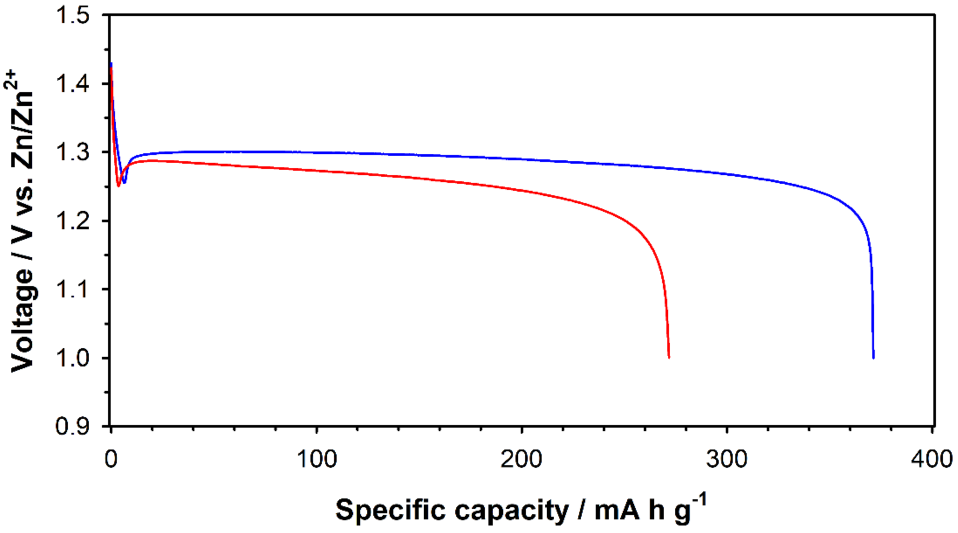

Figure S5. Voltage profiles of mild-acid Zn-MnO_2 cells at various current densities for the initial cycle (blue line: 7.7 mA g^{-1}, red line: 92.5 mA g^{-1}).

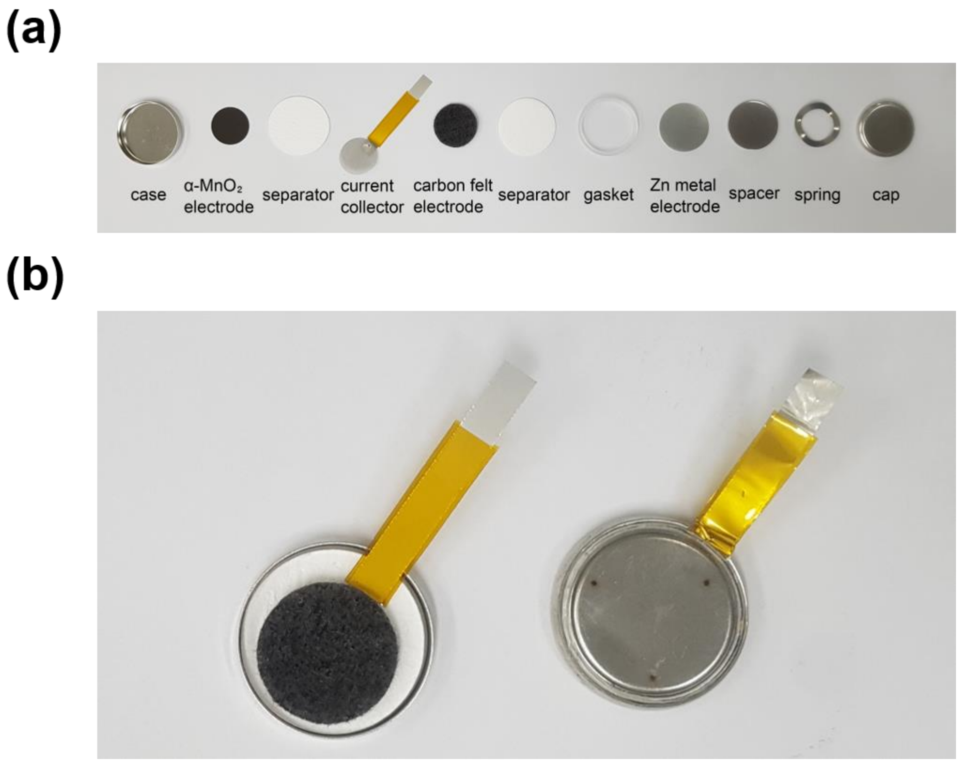

Figure S6. (a) Picture of all coin cell components for the two working electrodes system. (b) Picture of the coin cell assembly for the two working electrodes system.

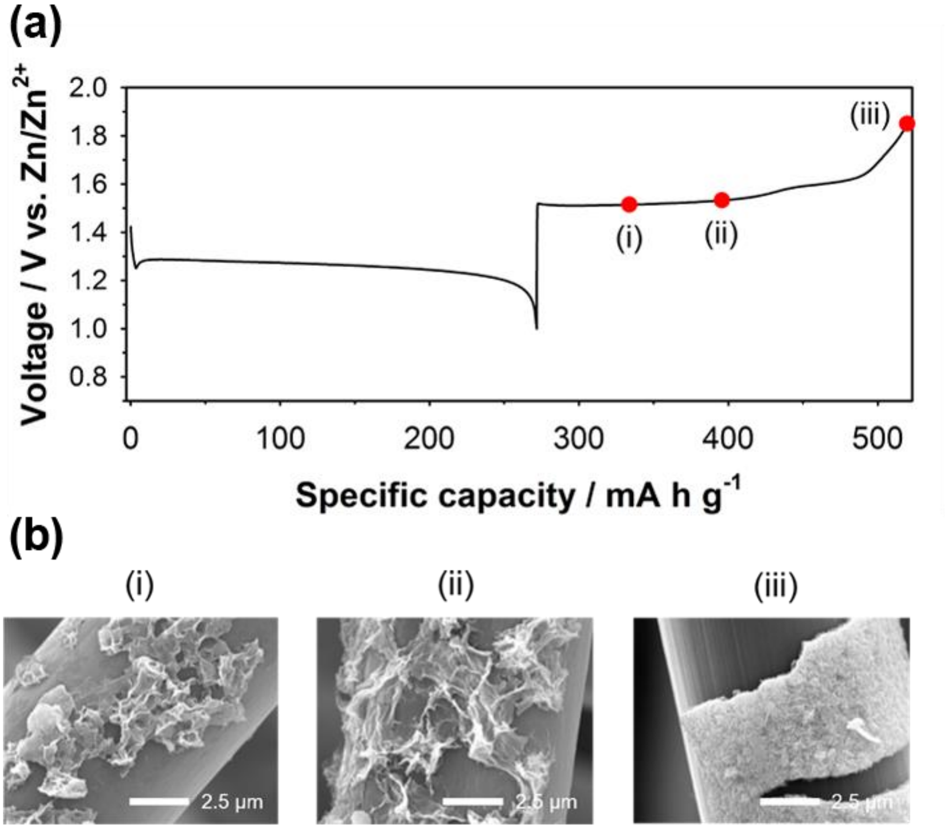

Figure S7. (a) Voltage profile of the Zn-MnO_2 cell for the first cycle. (b) Ex situ SEM images of the carbon felt electrode surface retrieved at various SOC states indicated in (a).

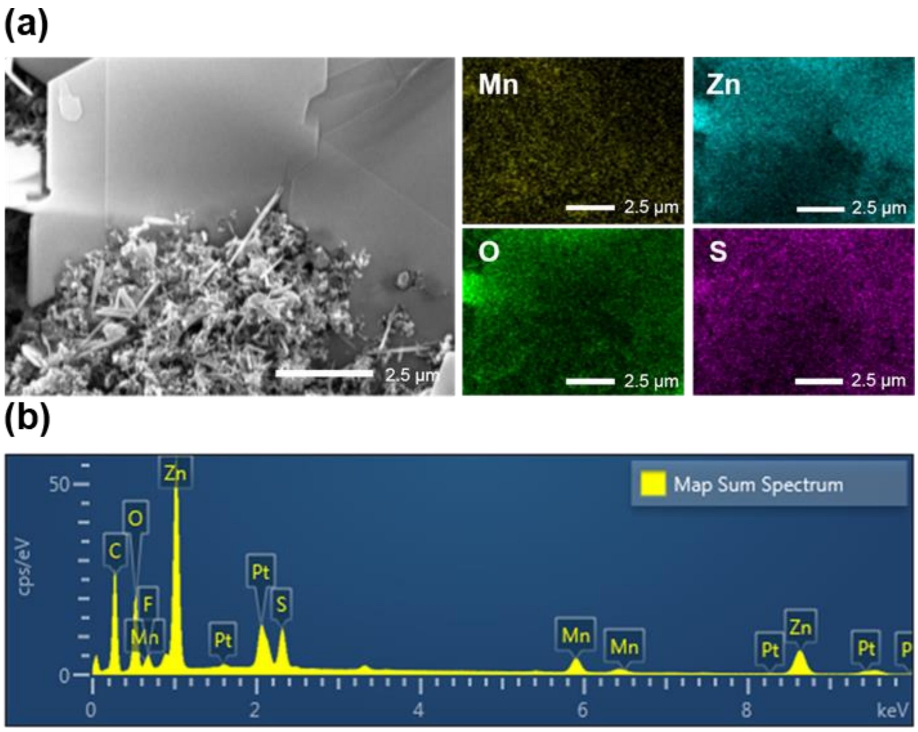

Figure S8. (a) Ex situ SEM and EDS mapping images and (b) the corresponding EDS spectrum of ZHS powders on the MnO_2 electrode surface after full discharge.

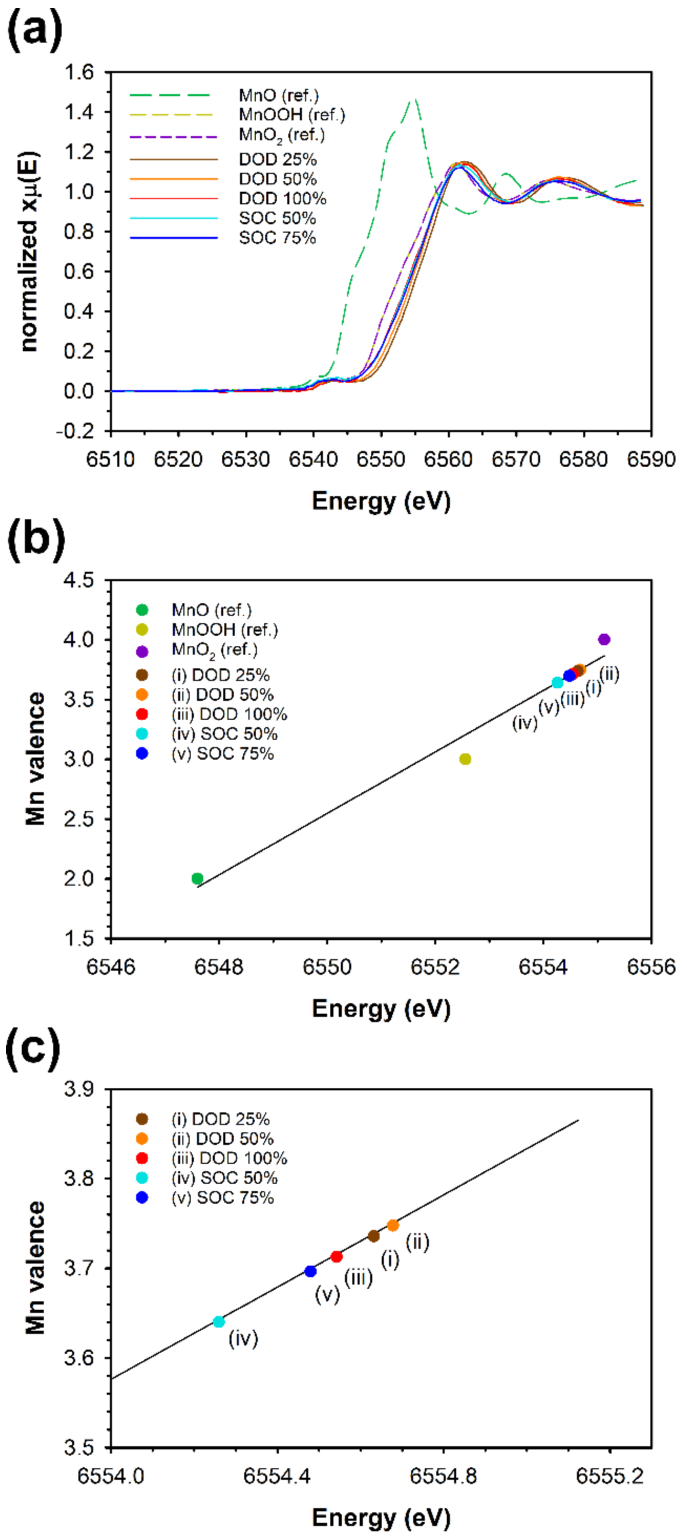

Figure S9. (a) Ex situ Mn-K edge XANES spectra of MnO_2 electrodes retrieved at various DOD and SOC states and the reference standards of MnO, MnOOH, and MnO_2. (b) Linear correlation between edge positions and oxidation states of Mn. (c) Enlarged display of (b) in a selected range of photon energy.

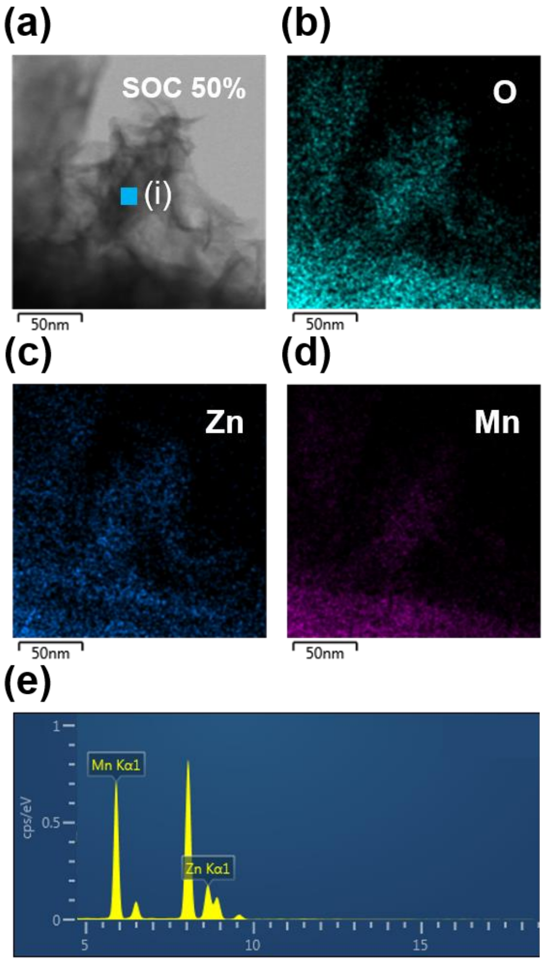

Figure S10. (a) Ex situ TEM and the corresponding EDS mapping images of the MnO_2 electrode retrieved at the SOC of 50%: (b) O, (c) Zn, and (d) Mn. (e) EDS spectrum obtained at point (i) in (a).

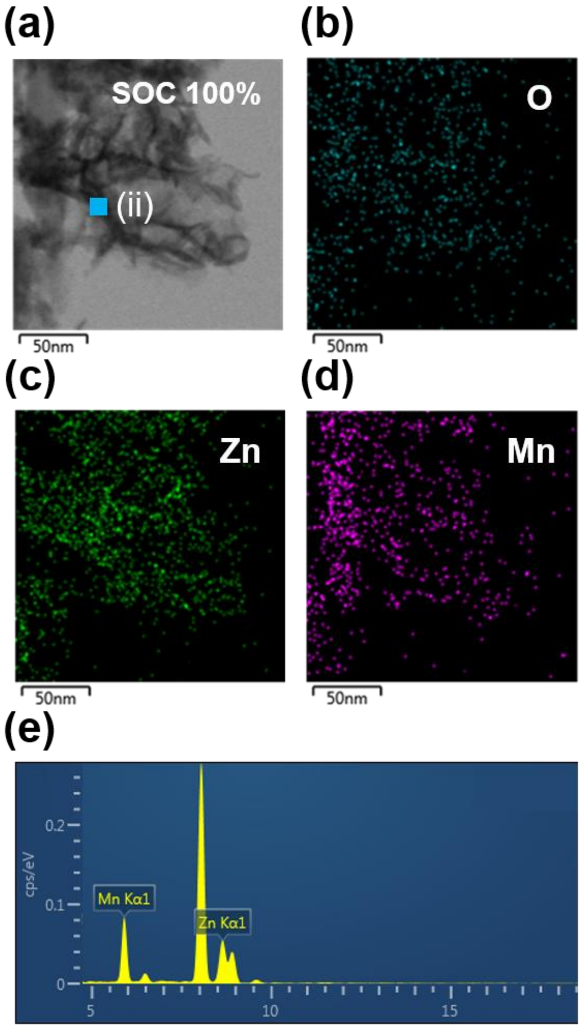

Figure S11. (a) Ex situ TEM and the corresponding EDS mapping images of the MnO_2 electrode retrieved at the SOC of 100%: (b) O, (c) Zn, and (d) Mn. (e) EDS spectrum obtained at point (ii) in (a).

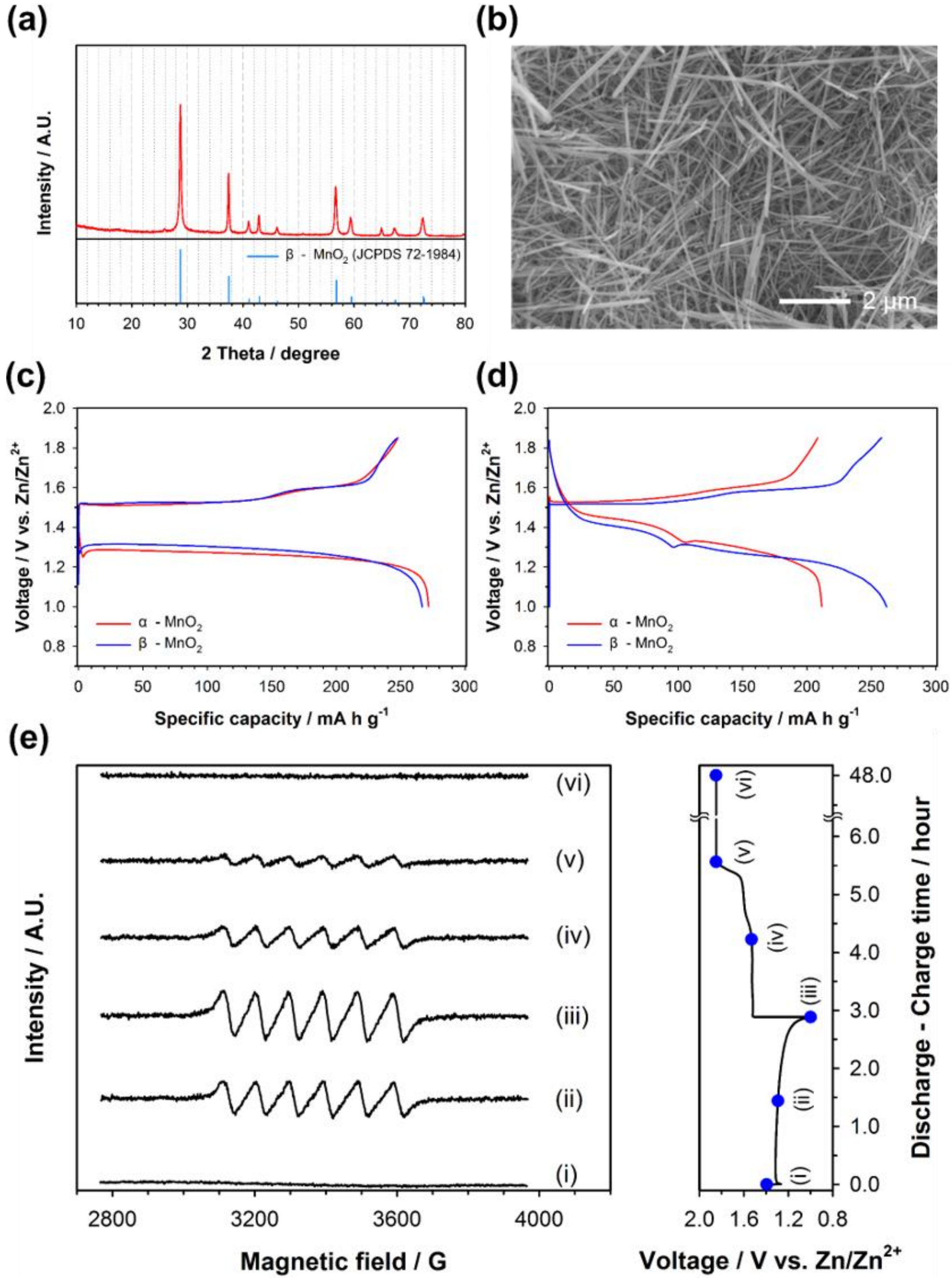

Figure S12. (a) XRD pattern and (b) FE-SEM image of β-MnO_2 nanorods. Voltage profile of α-MnO_2 and β-MnO_2 cathodes at a specific current of 92.5 mA g^{-1} for the (c) first and (d) second cycles. (e) Ex situ ESR spectra of the β-MnO_2 electrolytes retrieved at various discharge/charge states indicated in the voltage profile of (e).

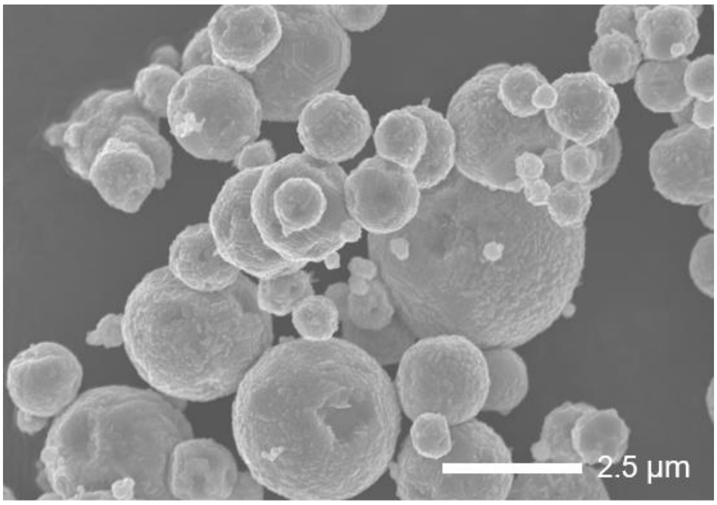

Figure S13. SEM image of Zn metal powders.

Table S1. The detailed specification of the 20 Ah-scale full cell.

Dimensions (cm^2)	25 × 30
Number of cathode sheets	15
Number of anode sheets	16
NP ratio	2.0
Cathode weight (mg cm^{-2})	11.1
Anode weight (mg cm^{-2})	5.5
Packing density of cathode (g ml^{-1})	1.1
Packing density of anode (g ml^{-1})	4
Electrolyte weight in the 20 Ah cell (g)	54.9
Total weight of the 20 Ah cell (g)	361
